# Impression Management and Interview and Job Performance Ratings: A Meta-Analysis of Research Design with Tactics in Mind

**DOI:** 10.3389/fpsyg.2017.00201

**Published:** 2017-02-15

**Authors:** Jessica A. Peck, Julia Levashina

**Affiliations:** Department of Management and Information Systems, Kent State UniversityKent, OH, USA

**Keywords:** impression management, employment interview, job performance, meta-analysis, research design

## Abstract

Impression management (IM) is pervasive in interview and job performance settings. We meta-analytically examine IM by self- and other-focused tactics to establish base rates of tactic usage, to understand the impact of tactics on interview and job performance ratings, and to examine the moderating effects of research design. Our results suggest IM is used more frequently in the interview rather than job performance settings. Self-focused tactics are more effective in the interview rather than in job performance settings, and other-focused tactics are more effective in job performance settings rather than in the interview. We explore several research design moderators including research fidelity, rater, and participants. IM has a somewhat stronger impact on interview ratings in lab settings than field settings. IM also has a stronger impact on interview ratings when the target of IM is also the rater of performance than when the rater of performance is an observer. Finally, labor market participants use IM more frequently and more effectively than students in interview settings. Our research has implications for understanding how different IM tactics function in interview and job performance settings and the effects of research design on IM frequency and impact.

## Introduction

Impression management (IM) is ubiquitous throughout the interview and during employment. Candidates and employees strive to put their best foot forward to impress employers (e.g., Barrick et al., 2009). IM is defined as conscious or unconscious, deceptive or authentic, goal directed behavior. Individuals behave or display props in an attempt to control the impressions others form of them in social interactions (e.g., Schlenker, 1980; Gardner and Martinko, 1988; Leary and Kowalski, 1990; Bozeman and Kacmar, 1997; Bolino et al., 2008, 2016). Researchers are examining a variety of experimental designs. However, single studies cannot assess the full magnitude of the relationship between IM tactics and ratings. Thus, the current paper positions a meta-analytical investigation of IM on interview and job performance ratings.

There are a few meta-analyses on IM and interview and performance outcomes (Higgins et al., 2003; Barrick et al., 2009; Levashina et al., 2014), but these studies each approach the IM and rating relationship from a specific lens leaving a gap in our overall understanding of IM. The most recent study by Levashina et al. (2014) examines these relationships in the context of the structured employment interview. They find self-promotion (*r* = 0.26) and other-focused (*r* = 0.13) tactics both impact structured interview ratings (Levashina et al., 2014). Higgins et al. (2003) analyze IM tactics through the lens of influence tactics and in many cases the dependent variable reflects a work outcome measure that includes combined interview ratings, performance ratings, and extrinsic measures of success. They find ingratiation has a stronger impact on ratings in lab studies (*r* = 0.33) than in field studies (*r* = 0.21) (Higgins et al., 2003). Barrick et al. (2009) meta-analytically examine self-presentation tactics of appearance, IM, and nonverbal and verbal behavior and the relationship with interview and performance outcomes. They find IM more strongly impacts interview ratings (*r* = 0.47) than job performance (*r* = 0.15) ratings. They also conclude self-presentation has a comparable impact on ratings in both the lab and the field. Yet, the impact of IM on ratings is slightly higher for field studies (*r* = 0.36) than lab studies (*r* = 0.30) and the impact of nonverbal and verbal behavior is higher for lab studies (*r* = 0.41) than field studies (*r* = 0.32) (Barrick et al., 2009). Each of these studies provide important information to advance the field forward, but the proliferation of IM research using various research design techniques necessitates an updated meta-analysis. The study herein establishes a base rate of IM in interview and job performance settings, explores the impact of self- and other-focused tactics on ratings, and examines research design factors that moderate the relationship between IM and interview and job performance outcomes.

## Theoretical background and hypothesis development

IM is a social influence process involving interactions between an actor, target, and environment (Goffman, 1959). Social influence theory suggests every social interaction involves one party trying to influence the other (Levy et al., 1998). Such motives are particularly invoked during situations in which an individual has the opportunity to develop an identity and obtain social and material outcomes. Social and material outcomes include obtaining a job in the case of an interview or obtaining a raise in the case of performance appraisal (Leary and Kowalski, 1990).

IM is most commonly categorized into self-focused (e.g., self-promotion) and other-focused (e.g., ingratiation) tactics. Self-focused tactics involve the applicant trying to promote perceptions of competence (Ellis et al., 2002). Interviewers form perceptions of the candidate during the interview and make attributions of competence. Self-focused tactics positively influence perceptions because the tactics limit the cognitive effort raters must go through to assess competence and instead directly provide attributional evidence for the individual's competence.

*Hypothesis 1a:* Self-focused tactics will be positively related to interview ratings.
*Hypothesis 1b:*Self-focused tactics will be positively related to job performance ratings.

Other-focused tactics are often used to elicit attraction, interpersonal liking, or perceptions of similarity (Ellis et al., 2002; Kristof-Brown et al., 2002), which are important influences on rating outcomes (Wayne et al., 1997).

*Hypothesis 2a:*Other-focused tactics will be positively related to interviewer ratings.
*Hypothesis 2b:*Other-focused tactics will be positively related to job performance ratings.

Interviews and job performance present unique situations for IM to occur. Interviews are shorter in nature and require less time for an individual to keep up impressions compared to ratings over a longer period of job performance. Also, asymmetric information during an interview allows candidates to engage in substantial IM because the interviewer does not have prior experience or knowledge of the candidate other than what is presented during the interview and in other selection measures (e.g., resumes, personality tests, etc.). However, supervisors have access to the candidate's history of behavior and can base ratings on job performance throughout the period rather than short term IM tactics. Further, interviews typically involve engaging with strangers, and job performance typically involves engaging with familiar people.

We posit that these situational differences between interviews and job performance lead to different impacts for self-promotion and ingratiation on interview and job performance ratings. Individuals are more prone to self-enhancement with strangers (Tice et al., 1995), and interviews typically take place between strangers rather than familiar others. IM as a goal directed behavior is desirable when it is beneficial to the actor and viewed as believable by the target (Schlenker, 2011). Self-focused tactics are beneficial in creating images of competence and believable if the interviewer is only relying on other selection measures and the current interview. Yet, these same self-focused tactics are not as believable or beneficial in generating positive job performance ratings. Supervisors are familiar with their employee's level of competence after exposure to performance over time, so self-promotion claims promoting competence are less beneficial and may even be unbelievable if such claims differ from the supervisor's own perceptions. Further, people generally become more modest over time as they get to know others, so the use of repeated self-promotive behaviors risks arrogance and can potentially damage relationships causing dislike (Schlenker, 2011). Prior research suggests self-promotion strategies have a negligible effect on supervisor liking (Wayne and Ferris, 1990) and in some cases a negative effect on supervisor liking, a precursor to career success (Judge and Bretz, 1994). On the other hand, ingratiatory tactics are suggested as more situationally appropriate in job performance settings than self-promotion tactics (Ferris et al., 1994). Prior research suggests other-focused tactics increase manager liking of subordinate and perceptions of similarity to the subordinate leading to increased performance ratings (Wayne et al., 1997). Researchers examining the effects of self-focused vs. other-focused tactics on interviews and job performance find other-focused tactics to have a greater impact on ratings during performance appraisals than during interviews (Kacmar and Carlson, 1999). In conclusion, we posit self-focused IM will more strongly impact interview rather than performance ratings. We also posit other-focused IM will more strongly impact performance ratings than interview ratings.

*Hypothesis 3a:*Self-focused tactics will be more strongly related to interview ratings than job performance ratings.
*Hypothesis 3b:*Other-focused tactics will be more strongly related to job performance ratings than to interview ratings.

### Research design moderators

We expect several important moderators related to research design to influence the relationship between IM and ratings. These moderators include fidelity of the research setting, whether the target of IM or an observer rates performance, and whether the participants are current labor market participants or students.

Researchers offer different opinions on the validity of experimental lab studies compared to high fidelity employment situations. Lab studies have similar levels of external validity to field studies if participants are placed in authentic situations that ensure psychological realism (Colquitt, 2008). Anderson et al. (1999) analyze meta-analyses of psychological research conducted in the lab vs. the field to determine the similarity between lab and field effect sizes. They correlate the effect sizes of the lab and field research for the same constructs and find a 0.73 correlation between effect sizes (Anderson et al., 1999). This relatively strong correlation is evidence of similar generalizability for lab and field studies in psychological research (Colquitt, 2008). However, the nature of the relationship between lab and field studies varies across particular literatures (Colquitt, 2008), which is the case for employment research.

Employment interview researchers often call into question the external validity of experimental lab studies compared to field studies, particularly due to the high-stakes nature of employment interviews and consequences of the rating (Jelf, 1999; Posthuma et al., 2002). The resulting experimental research on the impact of IM in lab and field studies has varying results. Higgins et al. (2003) find ingratiation has a higher impact on interview and job performance assessments in the lab rather than in the field. Barrick et al. (2009) find IM has a slightly higher impact on interview ratings in the field than in the lab, though they deem this difference insignificant. Barrick et al. (2009) also find verbal and non-verbal behavior has a higher impact in the lab than in the field. Despite these differing results, we expect IM has a higher impact on ratings in the lab rather than in the field based on the difference in accountability between lab and field settings.

Interviewers in high-stakes environments are accountable for the rating provided to the candidate as it often leads to employment. Interviewers in lab settings do not have the same level of accountability as the outcome of the rating generally has no consequences. Accountability research suggests individuals who are accountable make more accurate and consistent judgments (Ashton, 1992; Lerner and Tetlock, 1999). Raters who are judged based on ratee performance show higher levels of judgmental accuracy (Mero and Motowidlo, 1995). Therefore, interviewers in the field who are responsible for hiring a quality employee are more likely to focus on answers provided by the candidate rather than IM. This is true if other employees are aware of who is responsible for hiring the new employee and the interviewer is held accountable for the quality of the new hire. Individuals participating in lab settings are not responsible for the performance of the person who is fictitiously “hired” because this decision to “hire” has no actual consequences. Further, experimental research participants may assume any information is relevant to the experimental situation and the rating to be generated, so they are more likely to consider the effects of extraneous IM on interview ratings (Barrick et al., 2009). Therefore, we expect IM tactics to be more strongly related to interview ratings in lab settings than in field settings.

*Hypothesis 4:*IM tactics will be more strongly related to interview ratings in lab settings than in field settings.

The interviewer may both conduct the interview by asking the candidate questions and provide ratings of the candidate. Alternatively, there may be multiple individuals present in an interview with one person conducting the interview and another separate observer providing the ratings. Therefore, the target of IM may not always be the same person providing the rating of the individual. Therefore, this impacts the saturation of IM on interview ratings.

We view the moderating effect of the performance rater from two different perspectives. Limitations in human ability to cognitively process information suggests it is more difficult for individuals to go through the memory process of retrieving, transforming, and storing information with greater levels of information present (Wyer and Srull, 1981). Cognitive processing occurs sequentially and immediately during an interview evaluation so the more information necessary to transform, the higher the probability of information overload (Morgeson and Campion, 1997). Further, evidence from assessment center research suggests that as task complexity increases, rating accuracy decreases (Gaugler and Thornton, 1989). Therefore, interviewers actively asking questions, engaging with the individual, and providing an immediate rating are tasked with cognitively processing much more complex information than the observer who is just rating the individual. As such, it is more challenging for the target to separate out IM from job-related requirements, and we propose IM will be included in the ratings of the individual.

*Hypothesis 5a:*IM will be more strongly related to interview ratings when the target of IM has provided the rating than when an observer has provided the rating.

Alternatively, an active listener is able to perceive and eliminate extraneous IM that influences ratings more effectively than the observer. Active listening is conceptualized as having three elements including nonverbal involvement and communication, paraphrasing, and asking questions (e.g., Weger et al., 2010). Thus, an active listener is involved in the discussion, perceiving nonverbal and verbal signals and sending those same signals back to the candidate to show understanding and positive or negative acceptance of the communication. Brain connectivity patterns in active-response vs. passive-listening results show that active listening engages additional network connectivity elements of the brain associated with working memory and maintenance of attention (Wang and Holland, 2014). Such research indicates active listeners are more engaged in the conversation both cognitively and physiologically thus allowing them to perceive verbal and nonverbal IM information cues from the individual, send signals back to the individual about the acceptability of such information, and sort that information from actual candidate ratings to produce a rating that is less saturated with IM. Therefore, we propose active listeners will be able to filter IM from ratings whereas observers will not, making observer ratings of performance more saturated with IM.

*Hypothesis 5b:*IM will be more strongly related to interview ratings when the observer has provided the rating than when the target of IM has provided the rating.

Another research design factor previously unexplored is the impact of IM on ratings for research participants who are students compared to current labor market participants. We view this moderating effect from two separate theoretical perspectives.

First, we posit that IM is more strongly related to interview ratings for students than current labor market participants. Base rates for student faking behaviors, a form of deceptive IM, are established by Levashina and Campion (2007) across three studies. They find 85–99% of students engage in slight image creation, which is faking behavior they define as “to make an image of a good candidate for a job” (p. 1654). Also, 77–99% of students engage in ingratiation, which is faking behavior they define as “to gain favor with the interviewer to improve the appearance of a good candidate for the job.” This evidence suggests the use of such deceptive IM tactics is pervasive across student research participants, therefore the use of honest IM tactics used to convey an individual's actual qualifications is also likely pervasive.

Students are generally younger in age and have less work experience than current labor market participants. We draw from corporate fraud research that suggests younger individuals are more likely than older executives to engage in unethical or fraudulent activity (Daboub et al., 1995; Zahra et al., 2005). Further, there is an increased propensity to engage in illegal activities for more mobile executives with less work experiences compared to longer-tenured executives (Clinard, 1983), making length of work experience an important consideration. Also, it is possible that longer-tenured employees such as current labor market participants have more relevant work experience and skills, thus decreasing the need to use IM compared to students. Therefore, we posit that IM will be more strongly related to interview ratings for students than for current labor market participants.

*Hypothesis 6a:*IM will be more strongly related to interview ratings for students rather than current labor market participants.

Alternatively, current labor market participants are motivated to use IM techniques because the value of a job typically increases with more work experience and increased financial obligations. Current employees are, also, attuned to the perceived requirements of getting a new job, which is often advocated as “selling yourself” by career specialists (Ryan, 2016). There is also evidence that within organizations, longer-tenured employees engage in more IM. Women at senior levels of an organization engage in self-focused IM 70% of the time compared to junior women who engage in this behavior 30% of the time, according to a study focused on gender, age, and IM (Singh et al., 2002). In addition, most studies that involve current labor market participants are high-stakes situations that may lead to getting hired or receiving a positive performance review, so the current labor market participant is more motivated to engage in IM. Based on this notion, we argue that the relationship between IM tactics and ratings will be stronger for those currently engaged in the labor market rather than students.

*Hypothesis 6b:*IM will be more strongly related to interview ratings for current labor market participants than students.

## Methods

### Literature search

We reviewed articles over a 25 year period from 1990 to 2015. We chose this timeframe because several critical theoretical frameworks of IM were published around 1990 (Gardner and Martinko, 1988; Leary and Kowalski, 1990; Schlenker and Weigold, 1992), thus we expected IM research to proliferate after this time. We used the keyword search “*impression management*” to locate articles in the following journals: *Administrative Science Quarterly, Academy of Management Journal, Academy of Management Review, International Journal of Selection and Assessment, Journal of Applied Psychology, Journal of Management, Personnel Psychology, Organizational Behavior and Human Decision Processes, and Strategic Management Journal*.

### Inclusion criteria

Articles were included that contained an empirical analysis of IM tactics and either interview or job performance ratings with sample sizes and *r*-correlations or *d*-values that were used to convert the effect sizes into correlations. The resulting meta-analysis included 18 articles and 42 unique effect sizes that encompassed a sample size of 8,635.

### Description of variables

#### Impression management

Self-focused tactics included tactics such as exemplification, internal attributions, intimidation, professionalism, self-promotion, and supplication. Other-focused tactics included bargaining, favor rendering, appealing to higher authority, opinion conformity, other enhancement, ingratiation, and supervisor-focused tactics.

#### Interview rating

Interview rating was operationalized as an overall rating of interview performance. In a limited number of cases, interview rating was operationalized as person-job fit, hiring recommendation, post interview job beliefs and job offer expectancy.

#### Job performance

Job performance rating was operationalized as an evaluation of the employee's performance denoted as either task performance or promotability assessments. We pooled task performance and promotability assessments for sample size purposes after we analyzed means and correlations for each separately and determined they were similar.

#### Rating source

We separated studies according to whether ratings of interview or job performance were given by the target of the IM (interviewer or supervisor) or a third-party observer (colleague or observer).

#### Research fidelity

We separated studies into field vs. lab studies. We categorized studies as field studies if the study took place between an actual interviewer and job candidate or employee and supervisor and a job was at stake. We categorized studies as lab studies if no job was at stake and the study included a mock interview or experiment.

#### Research participants

Research participants were separated by whether the candidate was a student vs. already employed in the labor market.

### Meta-analytic procedures and artifact corrections

#### Non-independence of data

We followed Schmidt and Hunter (2014) recommendations for handling non-independence of data. Correlations between IM tactics and interview and job performance outcomes were recorded for each primary study. We converted *d*-values to *r*-correlations for studies that did not report *r*-correlations. After categorizing the studies by higher level groupings (i.e., other-focused, self-focused, etc.), many studies had multiple measures of the independent variable related to the dependent variable. In these cases, we computed composite correlations for the independent-dependent variable relationship to retain the independence of the sample. For studies that had IM outcomes with multiple measures of the interview or performance outcome variables, we selected the correlations that best represented the outcome variable of importance.

#### Unreliability corrections

We used Schmidt and Hunter (2014) artifact correction procedures for reliability. It was critical to correct for unreliability as it introduced measurement error that attenuated correlations (Schmidt and Hunter, 2014). Reliability was corrected individually per study using coefficient alpha values as this was the most commonly provided reliability information available from the primary study. We used the Spearman-Brown formula to compute composite reliabilities and used this reliability as the artifact correction for the composite (Schmidt and Hunter, 2014). The resulting corrected correlation was slightly overstated since Spearman-Brown reliability corrections assumed the components of each composite were orthogonal in their relationship to the outcome variable, which we knew with IM tactics and interview and job performance outcomes was likely not the case. In addition to correcting for unreliability in IM tactics, we also corrected for unreliability in interview and performance rating outcomes using reliabilities reported for each job and interview outcome measure in the primary study, which was an improvement over prior meta-analyses that did not correct the criterion using reliabilities reported by study.

## Results

Table 1 contains base rates of IM in interview and performance settings. IM is used more frequently overall in interviews (*M* = 4.42, *SDm* = 0.62) than performance settings (*M* = 3.80, *SDm* = 0.97). Other-focused tactics are used more frequently in interviews (*M* = 4.66, *SDm* = 0.68) than in performance settings (*M* = 2.68, *SDm* = 0.20), and self-focused tactics are used slightly more frequently in performance (*M* = 4.38, *SDm* = 0.66) than in interview settings (*M* = 4.30, *SDm* = 0.55).

**Table 1:** **Base rate of IM**.

**Analysis category**	***k***	***n***	***M***	***SDm***
Interview	17	2,097	4.42	0.62
Self-focused	10	1,399	4.30	0.55
Other-focused	7	698	4.66	0.68
Job performance	7	1,058	3.80	0.97
Self-focused	5	700	4.38	0.66
Other-focused	2	358	2.68	0.20

*k, number of means; n, number of subjects for analysis of means; M, sample weighted mean calculated on a 1–7 scale; SDm, sample-weighted standard deviation for mean*.

Table 2 contains the effects of IM tactics on ratings. Hypothesis 1 states self-focused tactics are positively related to (1a) interview ratings and (1b) performance ratings. We find support for hypothesis 1a as self-focused tactics are significantly related to interview ratings (*r*_*c*_ = 0.24, *p* < 0.05). We do not find support for hypothesis 1b as the relationship between self-focused tactics and performance ratings is not significant (*r*_*c*_ = 0.18, *n.s*.). Hypothesis 2 states other-focused tactics are positively related to (2a) interview ratings and (2b) performance ratings. We find support for both hypotheses 2a and 2b as other-focused tactics are significantly related to interview ratings (*r*_*c*_ = 0.17, *p* < 0.05) and job performance ratings (*r*_*c*_ = 0.25, *p* < 0.05).

**Table 2:** **Effects of IM on ratings**.

**Analysis category**	***k***	***n***	**Impact of IM on Ratings**				***% var***	**80% CV**		**95% CI**	
			***obs-r***	***SDr***	***r_c_***	***SDr_c_***		**Lower**	**Upper**	**Lower**	**Upper**
Interview	32	3,792	0.16	0.16	0.22	0.22	0.17	−0.07	0.50	0.14	0.29
Self-focused	20	2,515	0.17	0.18	0.24	0.25	0.13	−0.08	0.56	0.13	0.35
Other-focused	12	1,277	0.13	0.13	0.17	0.16	0.38	−0.03	0.37	0.08	0.26
Job performance	10	4,843	0.21	0.10	0.24	0.11	0.17	0.10	0.38	0.17	0.31
Self-focused	6	730	0.11	0.22	0.18	0.27	0.11	−0.17	0.52	−0.04	0.39
Other-focused	4	4,113	0.23	0.03	0.25	0.02	2.07	0.23	0.28	0.23	0.28

*k, number of correlations; n, number of subjects for analysis of correlations; obs-r, observed sample-weighted mean correlation; SDr, observed sample-weighted mean standard deviation; r_c_, sample-weighted mean correlation corrected for criterion unreliability; SDr_c_, sample-weighted mean standard deviation corrected for criterion unreliability; % var, percent variance explained by artifacts; CV, credibility value; CI, confidence interval*.

Hypothesis 3a states that self-focused IM is more strongly related to interview ratings than performance ratings. We find support for hypothesis 3a as self-focused tactics have a significant impact on interview ratings (*r*_*c*_ = 0.24, *p* < 0.05) and no significant impact on performance ratings (*r*_*c*_ = 0.18, *n.s.)*. Hypothesis 3b states that other-focused tactics are more strongly related to performance ratings than interview ratings. We find support for hypothesis 3b as other-focused tactics have a stronger impact on performance ratings (*r*_*c*_ = 0.25, *p* < 0.05) than interview ratings (*r*_*c*_ = 0.17, *p* < 0.05). Other-focused tactics are used more frequently in interview settings but more effectively in performance settings and self-focused tactics are used more frequently in performance settings but more effectively in interview settings. Results are presented in Tables 1, 2.

Hypothesis 4 states that IM is more strongly related to interview ratings in the lab than in the field. We find support for hypothesis 4. IM is used more frequently and is more strongly related to interview ratings in the lab (*M* = 4.48, *SDm* = 0.71, *r*_*c*_ = 0.24) vs. in the field (*M* = 4.36, *SDm* = 0.51, *r*_*c*_ = 0.18). However, the type of tactics used differs between the lab and the field. Self-focused tactics are used more frequently with more impact in the lab (*M* = 4.41, *SDm* = 0.72, *r*_*c*_ = 0.28) than the field (*M* = 4.19, *SDm* = 0.28, *r*_*c*_ = 0.16), yet other-focused tactics are used more frequently and with more impact in the field (*M* = 4.73, *SDm* = 0.67, *r*_*c*_ = 0.20) than in the lab (*M* = 4.59, *SDm* = 0.68, *r*_*c*_ = 0.15). Results are presented in Tables 3, 4.

**Table 3:** **Base rate of IM by research fidelity**.

**Analysis category**	***k***	***n***	***M***	***SDm***
Interview	17	2,097	4.42	0.62
Lab	8	1,035	4.48	0.71
Self-focused	5	674	4.41	0.72
Other-focused	3	361	4.59	0.68
Field	9	1,062	4.36	0.51
Self-focused	5	725	4.19	0.28
Other-focused	4	337	4.73	0.67
Job performance	7	1,058	3.80	0.97
Lab	1	87	3.36	0.77
Self-focused	1	87	3.36	0.77
Other-focused	–	–	–	–
Field	6	971	3.84	1.01
Self-focused	4	613	4.53	0.57
Other-focused	2	358	2.68	0.20

*k, number of means; n, number of subjects for analysis of means; M, sample weighted mean calculated on a 1–7 scale; SDm, sample-weighted standard deviation for mean. A dash (–) in the table indicates data was not available*.

**Table 4:** **Effects of IM on ratings by research fidelity**.

**Analysis category**	***k***	***n***	**Impact of IM on ratings**				***% var***	**80% CV**		**95% CI**	
			***obs-r***	***SDr***	***r_c_***	***SDr_c_***		**Lower**	**Upper**	**Lower**	**Upper**
Interview	32	3,792	0.16	0.16	0.22	0.22	0.17	−0.07	0.50	0.14	0.29
Lab	21	2,492	0.16	0.18	0.24	0.25	0.13	−0.09	0.56	0.13	0.34
Self-focused	14	1,671	0.19	0.20	0.28	0.27	0.11	−0.07	0.63	0.13	0.42
Other-focused	7	821	0.12	0.14	0.15	0.18	0.26	−0.08	0.38	0.01	0.28
Field	11	1,300	0.14	0.12	0.18	0.14	0.44	0.00	0.36	0.09	0.26
Self-focused	6	844	0.14	0.13	0.16	0.16	0.28	−0.04	0.37	0.04	0.29
Other-focused	5	456	0.15	0.09	0.20	0.09	1.46	0.09	0.32	0.13	0.28
Job performance	10	4,843	0.21	0.10	0.24	0.11	0.17	0.10	0.38	0.17	0.31
Lab	1	87	0.24	–	0.29	–	–	–	–	–	–
Self-focused	1	87	0.24	–	0.29	–	–	–	–	–	–
Other-focused	–	–	–	–	–	–	–	–	–	–	–
Field	9	4,756	0.21	0.10	0.24	0.11	0.16	0.10	0.38	0.17	0.31
Self-focused	5	643	0.09	0.23	0.16	0.28	0.10	−0.20	0.52	−0.09	0.41
Other-focused	4	4,113	0.23	0.03	0.25	0.02	2.07	0.23	0.28	0.23	0.28

*k, number of correlations; n, number of subjects for analysis of correlations; obs-r, observed sample-weighted mean correlation; SDr, observed sample-weighted mean standard deviation; r_c_, sample-weighted mean correlation corrected for criterion unreliability; SDr_c_, sample-weighted mean standard deviation corrected for criterion unreliability; % var, percent variance explained by artifacts; CV, credibility value; CI, confidence interval. A dash (–) in the table indicates data was not available*.

Hypothesis 5a states that there is a stronger relationship between IM tactics and interview ratings when the target of IM is also the rater of performance, while hypothesis 5b states that there is a stronger relationship between IM tactics and ratings when performance is rated by an observer rather than the target of IM. We find support for hypothesis 5a and not 5b. IM has a significant impact on ratings when the target of IM is also the rater of performance (*M* = 4.63, *SDm* = 0.54, *r*_*c*_ = 0.27). IM has no significant impact on ratings when the rater of performance is an observer, despite the frequency of IM use (*M* = 3.84, *SDm* = 0.58, *r*_*c*_ = 0.11). Results are presented in Tables 5, 6.

**Table 5:** **Base rate of IM by rater of performance and target of IM**.

**Analysis category**	***k***	***n***	***M***	***SDm***
Interview^a^	17	2,097	4.42	0.62
Rater: target of IM	11	1,488	4.63	0.54
Self-focused	7	1,011	4.50	0.39
Other-focused	4	477	4.90	0.68
Rater: observer	5	418	3.84	0.58
Self-focused	2	197	3.51	0.66
Other-focused	3	221	4.14	0.25
Job performance^a^	7	1,058	3.80	0.97
Rater: target of IM	6	959	3.78	1.02
Self-focused	4	601	4.44	0.69
Other-focused	2	358	2.68	0.20
Rater: observer	–	–	–	–
Self-focused	–	–	–	–
Other-focused	–	–	–	–

*k, number of means; n, number of subjects for analysis of means; M, sample weighted mean calculated on a 1–7 scale; SDm, sample-weighted standard deviation for mean. A dash (–) in the table indicates data was not available*.

a *Two studies that met these criteria were missing data and could not be included in the detailed analysis*.

**Table 6:** **Effects of IM on ratings by rater of performance and target of IM**.

**Analysis category**	***k***	***n***	**Impact of IM on ratings**				***% var***	**80% CV**		**95% CI**	
			***obs-r***	***SDr***	***r_c_***	***SDr_c_***		**Lower**	**Upper**	**Lower**	**Upper**
Interview^a^	32	3,792	0.16	0.16	0.22	0.22	0.17	−0.07	0.50	0.14	0.29
Rater: target of IM	23	2,658	0.20	0.15	0.27	0.19	0.24	0.03	0.51	0.19	0.35
Self-focused	15	1,777	0.22	0.14	0.30	0.19	0.24	0.06	0.54	0.21	0.40
Other-focused	8	881	0.15	0.15	0.21	0.17	0.30	−0.02	0.43	0.09	0.33
Rater: observer	8	943	0.08	0.16	0.11	0.26	0.13	−0.21	0.44	−0.06	0.29
Self-focused	4	547	0.09	0.21	0.13	0.33	0.06	−0.29	0.56	−0.19	0.46
Other-focused	4	396	0.08	0.04	0.09	0.05	4.37	0.03	0.15	0.04	0.13
Job performance^a^	10	4,843	0.21	0.10	0.24	0.11	0.17	0.10	0.38	0.17	0.31
Rater: target of IM	8	1,019	0.08	0.11	0.16	0.16	0.30	−0.05	0.37	0.05	0.27
Self-focused	5	631	0.03	0.11	0.09	0.16	0.32	−0.12	0.29	−0.05	0.23
Other-focused	3	388	0.15	0.05	0.28	0.06	1.86	0.20	0.36	0.21	0.35
Rater: observer	–	–	–	–	–	–	–	–	–	–	–
Self-focused	–	–	–	–	–	–	–	–	–	–	–
Other-focused	–	–	–	–	–	–	–	–	–	–	–

*k, number of correlations; n, number of subjects for analysis of correlations; obs-r, observed sample-weighted mean correlation; SDr, observed sample-weighted mean standard deviation; r_c_, sample-weighted mean correlation corrected for criterion unreliability; SDr_c_, sample-weighted mean standard deviation corrected for criterion unreliability; % var, percent variance explained by artifacts; CV, credibility value; CI, confidence interval. A dash (−) in the table indicates data was not available*.

a *Three studies that met these criteria were missing data and could not be included in the moderation analysis*.

Hypothesis 6a states that there is a stronger relationship between IM tactics and ratings when the research participants are students rather than current labor market participants. On the other hand, hypothesis 6b states that there is a stronger relationship between IM tactics and ratings when current labor market participants are research participants rather than students. We find support for hypothesis 6b and not 6a. There is a stronger relationship between IM tactics and ratings for current labor market participants than students. IM tactics were used slightly more frequently by current labor market participants (*M* = 4.37, *SDm* = 0.18, *r*_*c*_ = 0.36) than students (*M* = 4.31, *SDm* = 0.68, *r*_*c*_ = 0.15) and considerably more effectively. Results are presented in Tables 7, 8.

**Table 7:** **Base rate of IM by research participant**.

**Analysis category**	***k***	***n***	***M***	***SDm***
Interview^a^	17	2,097	4.42	0.62
Labor market participant	4	562	4.37	0.18
Self-focused	3	457	4.45	0.11
Other-focused	1	105	4.05	0.00
Student	12	1,351	4.31	0.68
Self-focused	7	942	4.22	0.66
Other-focused	5	409	4.52	0.69
Job performance	7	1,058	3.80	0.97
Labor market participant	7	1,058	3.80	0.97
Self-focused	5	700	4.38	0.66
Other-focused	2	358	2.68	0.20
Student	–	–	–	–
Self-focused	–	–	–	–
Other-focused	–	–	–	–

*k, number of means; n, number of subjects for analysis of means; M, sample weighted mean calculated on a 1–7 scale; SDm, sample-weighted standard deviation for mean. A dash (–) in the table indicates data was not available*.

a *One study that met this criteria was missing data and could not be included in the detailed analysis*.

**Table 8:** **Effects of IM on ratings by research participant**.

**Analysis category**	***k***	***n***	**Impact of IM on ratings**				***% var***	**80% CV**		**95% CI**	
			***obs-r***	***SDr***	***r_c_***	***SDr_c_***		**Lower**	**Upper**	**Lower**	**Upper**
Interview^a^	32	3,792	0.16	0.16	0.22	0.22	0.17	−0.07	0.50	0.14	0.29
Labor market participant	10	1,162	0.26	0.13	0.36	0.17	0.30	0.15	0.58	0.26	0.47
Self-focused	8	938	0.29	0.12	0.39	0.18	0.28	0.16	0.61	0.27	0.51
Other-focused	2	224	0.16	0.11	0.25	0.06	2.38	0.17	0.33	0.16	0.33
Student	21	2,446	0.10	0.16	0.15	0.22	0.18	−0.14	0.43	0.05	0.24
Self-focused	12	1,577	0.10	0.17	0.15	0.24	0.13	−0.16	0.46	0.01	0.29
Other-focused	9	869	0.11	0.14	0.14	0.18	0.32	−0.09	0.37	0.02	0.26
Job performance	10	4,843	0.21	0.10	0.24	0.11	0.17	0.10	0.38	0.17	0.31
Labor market participant	10	4,843	0.21	0.10	0.24	0.11	0.17	0.10	0.38	0.17	0.31
Self-focused	6	730	0.11	0.22	0.18	0.27	0.11	−0.17	0.52	−0.04	0.39
Other-focused	4	4,113	0.23	0.03	0.25	0.02	2.07	0.23	0.28	0.23	0.28
Student	–	–	–	–	–	–	–	–	–	–	–
Self-focused	–	–	–	–	–	–	–	–	–	–	–
Other-focused	–	–	–	–	–	–	–	–	–	–	–

*k, number of correlations; n, number of subjects for analysis of correlations; obs-r, observed sample-weighted mean correlation; SDr, observed sample-weighted mean standard deviation; r_c_, sample-weighted mean correlation corrected for criterion unreliability; SDr_c_, sample-weighted mean standard deviation corrected for criterion unreliability; % var, percent variance explained by artifacts; CV, credibility value; CI, confidence interval. A dash (–) in the table indicates data was not available*.

a *One study that met this criterion was missing data and could not be included in the moderation analysis*.

## Discussion

The purpose of this meta-analysis was to establish base rates of IM in interview and job performance settings, explore the impact of self- and other-focused tactics on ratings, and examine research design factors that moderate the relationship between IM and interview ratings. We found strong evidence overall that IM saturated interview and performance ratings. Further, research design proved to be an important consideration. IM was used slightly more frequently and with slightly more impact in the lab than in the field. However, these results differed substantially when examining the specific IM tactic. In particular, self-focused tactics had a much higher impact on ratings in the lab than in the field while other-focused tactics had a slightly stronger impact on ratings in the field than in the lab. Therefore, researchers should be cognizant of the type of IM under investigation and how the research design may affect the frequency and impact of IM on their ratings.

Targets of IM who also provided performance ratings had stronger IM-rating relationships than observers who provided performance ratings. This result supported the notion that individuals who actively asked questions, engaged with the individual, and provided an immediate rating were tasked with high levels of cognitive processing that made it challenging for the target to separate out IM from job-related rating requirements. Therefore, IM was included in the ratings of the individual.

Future research should examine whether IM is considered to be a contamination variable or job-related. If IM is assumed to be a contamination variable, then the accuracy of ratings may be improved by having one person directly ask questions and another responsible for providing a performance assessment. If IM is assumed to be job-related, then it may be appropriate that the interviewer is including the IM in ratings.

We also found support for the notion that current labor market participants used IM slightly more frequently and significantly more effectively than students. This suggested that perhaps IM was a learned skill. More experienced workers were better able to identify when IM use was appropriate and applied it with relatively similar frequency as students but yielded more effective results. Although not hypothesized, this result coincided with results in Tables 1, 2 that suggested IM was used less frequently overall in job performance than interviews but had a stronger impact on ratings. Current labor market participants rated on job performance were able to use IM tactics more adeptly and effectively.

Researchers should be mindful of these differences between current labor market participants and students. Further, employers interviewing entry-level employees vs. long-tenured employees may want to consider the differential impact of various IM tactics.

### Limitations

Our study is not without limitations despite the interesting results. First, we are not able to perform moderation analysis on research design and job performance due to the lack of primary studies of IM in a job performance setting. We encourage additional primary studies focused on IM tactics and job performance. We know the effects of IM tactics on ratings outcomes differ between interviews and job performance settings, so there may be additional differences in how the impact of IM on ratings is altered by research design in job performance settings that cannot be assumed just by looking at the impact on interview ratings.

Second, there are certain methodological limitations based on availability of information from primary studies. Despite our rigorous use of individual level study artifact corrections, we use coefficient alpha as our reliability estimate, which does not include transient error and thus under corrects for measurement error if transient error is present (Schmidt and Hunter, 2014). Also, we use Spearman-Brown as our composite reliability correction, which may overestimate reliability estimates (Schmidt and Hunter, 2014).

Third, several of our credibility intervals are quite large indicating substantial variation in parameter estimates across primary studies. These intervals suggest moderators of the relationships. We address many critical moderators in this study by looking at interview and performance settings separately, splitting IM tactics into self- and other-focused, and analyzing research design factors, but other moderators of these relationships should be explored in the future.

## Conclusion

This study helps further elucidate the frequency and impact of IM on interview and performance ratings. Further, research design factors such as research fidelity, rater, and research participants have important effects on the impact of IM on ratings. Therefore, adjustments to these factors may strengthen or attenuate the relationship between IM and ratings, which is useful to future researchers and practitioners.

## Author contributions

JP, JL were both engaged in substantial contributions to the conception and design of the work, drafting and revising the work for important intellectual content, final approval of the version to be published, and agree to be accountable for all aspects of the work.

### Conflict of interest statement

The authors declare that the research was conducted in the absence of any commercial or financial relationships that could be construed as a potential conflict of interest.
